# The Impact of Detoxification Costs and Predation Risk on Foraging: Implications for Mimicry Dynamics

**DOI:** 10.1371/journal.pone.0169043

**Published:** 2017-01-03

**Authors:** Christina G. Halpin, John Skelhorn, Candy Rowe, Graeme D. Ruxton, Andrew D. Higginson

**Affiliations:** 1 Centre for Behaviour and Evolution, Institute of Neuroscience, Newcastle University, Newcastle upon Tyne, United Kingdom; 2 School of Biology, University of St. Andrews, St Andrews, United Kingdom; 3 Centre for Research in Animal Behaviour, College of Life and Environmental Sciences, University of Exeter, Exeter, United Kingdom; University of Sussex, UNITED KINGDOM

## Abstract

Prey often evolve defences to deter predators, such as noxious chemicals including toxins. Toxic species often advertise their defence to potential predators by distinctive sensory signals. Predators learn to associate toxicity with the signals of these so-called aposematic prey, and may avoid them in future. In turn, this selects for mildly toxic prey to mimic the appearance of more toxic prey. Empirical evidence shows that mimicry could be either beneficial (‘Mullerian’) or detrimental (‘quasi-Batesian’) to the highly toxic prey, but the factors determining which are unknown. Here, we use state-dependent models to explore how tri-trophic interactions could influence the evolution of prey defences. We consider how predation risk affects predators’ optimal foraging strategies on aposematic prey, and explore the resultant impact this has on mimicry dynamics between unequally defended species. In addition, we also investigate how the potential energetic cost of metabolising a toxin can alter the benefits to eating toxic prey and thus impact on predators’ foraging decisions. Our model predicts that both how predators perceive their own predation risk, and the cost of detoxification, can have significant, sometimes counterintuitive, effects on the foraging decisions of predators. For example, in some conditions predators should: (i) avoid prey they know to be undefended, (ii) eat more mildly toxic prey as detoxification costs increase, (iii) increase their intake of highly toxic prey as the abundance of undefended prey increases. These effects mean that the relationship between a mimic and its model can qualitatively depend on the density of alternative prey and the cost of metabolising toxins. In addition, these effects are mediated by the predators’ own predation risk, which demonstrates that, higher trophic levels than previously considered can have fundamental impacts on interactions among aposematic prey species.

## Introduction

Prey animals often evolve defences, such as toxic or noxious chemicals, and advertise this to potential predators, using distinctive coloration, sounds, or scents (e.g. [1,2,3]. The combination of a toxin with a salient warning signal is known as aposematism [4]. Predators are able to learn to associate the warning signal with the toxicity in order to regulate their intake of toxins (e.g. [5,6–10]). Sympatric aposematic species sometimes share the same visual warning signal: they are Müllerian mimics [11]. Müllerian mimics are thought to mutually benefit by sharing the cost of educating predators, resulting in fewer individuals of both species being killed by naïve predators ([11], and see [12] for an overview of empirical evidence). However, if the mimics contain unequal amounts of toxin, the less defended species may be detrimental to the survival of the more toxic species; so-called ‘quasi-Batesian’ mimicry [13,14]. Laboratory experiments have found mixed evidence for quasi-Batesian mimicry dynamics between unequally defended mimics [15–18]. Here, we propose that these mixed results are found because the physiological or psychological states of predators [19–22]vary across contexts, and this in turn will alter predator foraging decisions, which could in turn alter the relationship between the two mimetic prey types.

Avian predators are able to learn about the toxin content of prey and can use this knowledge to accurately manage their toxin burden (the amount of toxin in their body) ([7,8,15,19,23,24,25]). However, prey in the wild use a range of different toxins (e.g. [26,27,28]) at concentrations that can vary both within and between prey species (e.g. [29,30–33]) and it is likely that any given toxin will affect different predators in different ways [34–37]. Yet, we do not know how important the metabolic costs associated with ingesting different toxins are in the predators’ decisions to eat toxic prey [38], which could vary across species [39,40]. A better understanding of the influence of these costs could potentially help us better understand the equivocal findings surrounding mimicry dynamics, in particular those of quasi-Batesian mimicry [15–18].

Foraging predators are usually themselves at heightened risk of predation [41], due to being less vigilant, or more frequently encountering or being detected by predators through movement (e.g. [42,43,44]). Therefore, a foraging predator must weigh mortality risk against the need to meet its energetic and nutritional requirements. This risk can affect many aspects of foraging behaviour, such as the choice of foraging sites and diurnal foraging patterns [43,45,46]. The trade-off can also directly impact on predator body mass by affecting levels of energy reserves [45,47]: whilst storing fat can be beneficial and provide a buffer for when food is scarce, it can also make predators less manoeuvrable and so more susceptible to predation themselves (e.g., [48,49,50]). Intriguingly, body mass and fat stores also appear to affect predators’ decisions to eat toxic prey [23,24]. This means that by affecting state and subsequently state-dependent foraging decisions, a predator’s own perceived predation risk could influence the evolution of prey defences. However, we don’t know how the foraging decisions of predators on toxic prey might be affected by their need to acquire nutrients whilst at the same time avoiding predation.

Here, we examine how the food–predation trade-off affects predators’ optimal foraging strategies on aposematic prey, and explore the resultant impact these strategies have on the mimicry dynamics between unequally defended sympatric aposematic species. Whilst previous theoretical models have highlighted the importance of physiological state on predators’ decisions and the evolutionary dynamics of defence strategies [20,22,38,51], ours considers how tri-trophic interactions (between predators, their predators and their prey) could influence the evolution of prey defences. Since ingesting toxins can be energetically costly to predators (e.g. [52]), and we know that the intake of toxins can affect the dietary decisions of birds [39,40], we also investigate how the cost of metabolising a toxin can alter the benefits to eating toxic prey and impact on predators’ foraging decisions. Our model predicts that how predators perceive their own predation risk, as well as the cost of detoxification, can have a significant, yet sometimes counterintuitive, effect on the foraging decisions of a predator, and in turn on the survival of toxic prey in the environment. In particular, the relationship between a mildly toxic mimic and its more highly toxic model is predicted to qualitatively depend on the density of alternative prey and the cost of metabolising toxins.

## The Model

We characterize predators as two state variables: energy reserves *R* and current toxin burden *D*. We assume that predators can die from starvation, by being eaten by their own predators, or by exceeding their maximum tolerated toxin burden. Increasing *R* reduces the long-term risk of starvation but increases the risk of predation per unit time. Increasing *D* also carries risks, since predators may have to avoid potentially defended prey when they have a high toxin burden (because an excessive toxin dose can be fatal), which means a higher risk of starving when energy reserves are low. However, the model predicts that mortality from toxin overdose is extremely rare, which reflects what we know from empirical findings that avian predators are able to accurately manage their toxin burden (e.g. [7,8,15,19,23,24,25]).

We model foraging in an environment that contains three prey types denoted by α, β, and γ. Prey α and β are aposematic, in that they are conspicuous and chemically defended, but they contain different amounts of the same toxin: α is mildly toxic and β is highly toxic. Prey γ has a cryptic appearance but is otherwise undefended and palatable. At each time-step the predator stochastically encounters either nothing or a prey type. The probabilities of each encounter are determined by the availability of the three prey types (*f*_α_, *f*_β_, *f*_γ_) which is the product of the abundance and the conspicuousness of each type. Therefore, although cryptic prey are much more abundant than aposematic prey, they are more difficult to find and so have availability on the same order of magnitude. We assume that the predator has perfect knowledge of the encounter rates of prey. We also assume that predator abundance changes over a much slower timescale than prey populations, as is commonly the case in natural systems, and that the predators adjust their strategies within lifetimes to the various prey distributions.

As they forage, predators pay a cost ρ per unit time to meet their energetic needs. All prey types offer energetic resources for the predator: ingesting an individual of type *p* gains the predator *r*_*p*_ resources (where *p* is α, β or γ). If the predator ingests an α or β individual, then its toxin burden increases by *d*_*α*_ or *d*_*β*,_ respectively. When the predator has a positive toxin burden it detoxifies one unit of toxin per unit time, which incurs an extra metabolic cost per unit time, κ.

The increasing vulnerability to predators of animals as their fat stores increase [49] implies that the risk of predation depends on the level of reserves. We assume that the risk of predation per time step *M*(*R*) is given by

$$M(R)=\mu \left(\frac{1}{2}+\frac{2R}{R_{\max}}\right)$$ (1)

where *μ* is proportional to the density of predators in the environment. The second term in parentheses implies that predation is mass-dependent; specifically, an animal with reserves (*R = R*_*max*_) is five times as likely to be killed by a predator as an animal with no reserves (*R* = 0). For computational reasons we must choose *R*_*max*_ but preliminary explorations showed that the model’s predictions and the conclusions drawn were not dependent on the parameter value.

There are five options for choosing which prey to eat (δ):

$$\delta =\left\{ \begin{array}{l} 0\;\text{eat no prey}\\ 1\;\text{refuse to eat both}\;\alpha \;\text{and}\;\beta \\ 2\;\text{refuse to eat}\;\alpha \;\text{but eat}\;\beta \\ 3\;\text{refuse to eat}\;\beta \;\text{but eat}\;\alpha \\ 4\;\text{eat both}\;\alpha \;\text{and}\;\beta \end{array}\right.$$ (2)

In all decisions except δ = 0 the predator eats γ. The only reason for a predator to avoid eating any prey (δ = 0) is that increasing *R* will increase the risk of predation more than it will decrease the long-term risk of starvation, and so thereby decrease expected survival.

We assume that in some conditions prey types α and β are perfect mimics, meaning that they are identical in appearance and the predator cannot distinguish between them. The ability of a predator to distinguish between them is determined by the parameter ε, which takes the values zero (perfectly distinguishable) or one (perfectly indistinguishable). Note that the cryptic prey γ are always assumed to be distinguishable from both α and β.

Using established dynamic programming methods described in S1 Appendix, we find the optimal option (δ, equation 2) for every integer combination of predator states (*R*, *D)*. We then run stochastic simulations with *N* predators following the optimal strategy to find the number of times that each prey type survives (*S*_*p*_) or does not survive (*K*_*p*_) an encounter with a predator. This allows us to calculate the probability of survival for each prey type. Baseline parameter values are shown in Tables 1 and 2. These were obtained by exploring parameter space to find where predator behaviour was realistic (e.g. discrimination of prey types, appropriate energy storage for small passerine, probability of survival, etc.) and comparable to empirical findings [15]. We simulate four conditions that are the same as those used in experiments aimed at understanding the mimicry dynamics between unequally defended prey (e.g. [15,16]): (1) Mildly defended prey alone (*f*α>0, *f*_β_ = 0, *f*_γ_>0); (2) Highly defended prey alone (*f*_α_ = 0, *f*_β_>0, *f*_γ_>0); (3) Distinguishable mildly and highly defended prey (*f*_α_>0, *f*_β_>0, *f*_γ_>0, ε = 0); (4) Indistinguishable mildly and highly defended prey (*f*_α_>0, *f*_β_>0, *f*_γ_>0, ε = 1). These four conditions allow us to compare what the benefits are for a defended prey type of being sympatric with another defended prey type (comparing conditions 1 and 2 with condition 3), and what the costs and benefits of being mimetic are for each sympatric prey type (comparing condition 3 with condition 4).

**Table 1:** Parameters of the predator and the values explored.

	Symbol	Value
Number of predators	*N*	1000
Number of time steps	*T*	500
Maximum energy level	*R*_*max*_	200
Maximum toxin level	*D*_*max*_	50
Mortality risk to predators	μ	10^−6^
Basal metabolic cost	ρ	3.5
Error rate for distinguishing models	ε	0 or 1
Cost of metabolising toxin	κ	0 or 1

**Table 2:** Parameters of the prey and the values explored.

(b)	Mimic *α*	Model *β*	Alternative *γ*
**Availability** *f*	0.2	0.2	0.1, 0.2, 0.3
**Energy** *r*	10	10	10
**Toxin** *d*	5	10	0

Note that lower-case Roman symbols are used for prey, upper-case Roman symbols for predator state, Greek symbols for other parameters.

## Results

### Optimal foraging strategies of the predator

**Fig 1:**
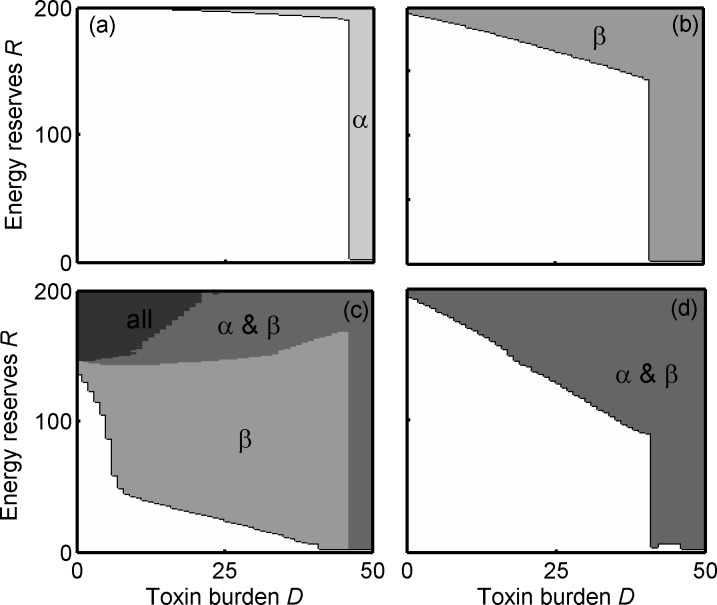
Optimal foraging strategies when detoxification is not costly. Optimal foraging strategy for predators, shown as which prey types are rejected as a function of energy reserves *R* and toxin burden *D* for the four treatments: (a) mildly defended prey, α alone; (b) highly defended prey, β alone; (c) α and β both present and visually distinguishable; (d) α and β both present and perfect mimics. Here, there is no detoxification cost (κ = 0) and alternative prey are at intermediate availability (γ = 0.2). The shaded areas show the states where: the predators reject all prey including alternative prey (black); reject only the mildly defended prey (pale grey); reject only the highly defended prey (intermediate grey) reject both defended prey (dark grey). In the white areas, all prey are accepted.

**Fig 2:**
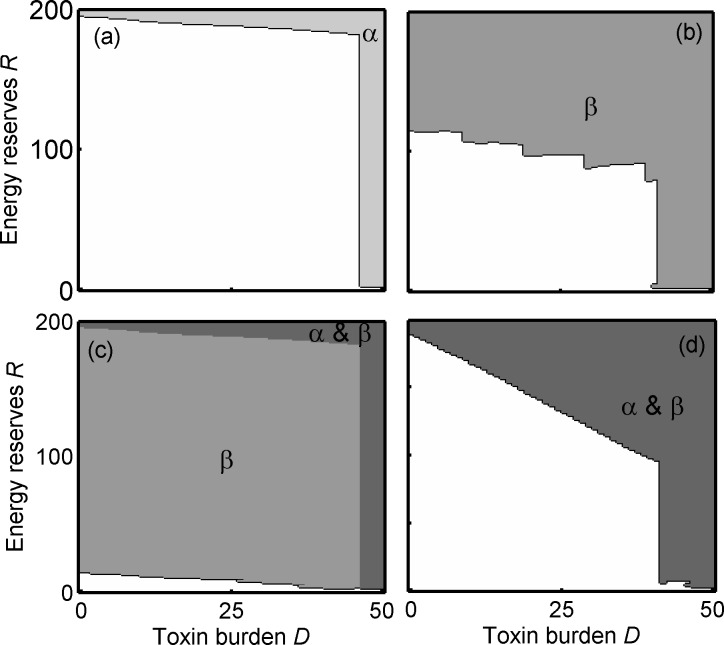
Optimal foraging strategies when detoxification is costly. Optimal foraging strategy for predators, shown as which prey types are rejected as a function of energy reserves *R* and toxin burden *D* for the four treatments: (a) mildly defended prey, α alone; (b) highly defended prey, β alone; (c) α and β both present and visually distinguishable; (d) α and β both present and perfect mimics. Here, there is a detoxification cost (κ = 1) and alternative prey are at intermediate availability (γ = 0.2). The shaded areas show the states where: the predators reject all prey including alternative prey (black); reject only the mildly defended prey (pale grey); reject only the highly defended prey (intermediate grey) reject both defended prey (dark grey). In the white areas, all prey are accepted.

We initially describe the optimal strategies for a predator in each of our four conditions when an intermediate number of alternative prey (γ = 0.2) are also available, and the energetic cost to predators associated with metabolizing toxins is either negligible (κ = 0; Fig 1) or significant (κ = 1; Fig 2). We will then consider what implications these foraging strategies have on prey survival and the dynamics between two unequally toxic prey species that are either visually mimetic or not.

### A single defended prey type should be rejected at high toxin burden and high energy reserves, irrespective of the defence level

When there is only one defended prey type in the system, predator strategies are similar whether the defended prey type is mildly toxic (α) or highly toxic (β): (i) when a predator has a high toxin burden it should reject the defended prey type, whether there is a detoxification cost (Fig 2A and 2B) or not (Fig 1A and 1B), since eating another defended prey could be fatal, and (ii) when a predator has very high energy reserves, it should reject the defended prey type at most toxin burdens (Figs 1A, 1B, 2A and 2B). This is because the risk of starvation is low and increasing energy reserves will increase the predator’s own risk of being predated. As a predator’s toxin burden increases the energy reserve levels at which defended prey should be rejected decreases (Figs 1A, 1B, 2A and 2B).

There are some notable small differences in the optimal response of predators faced with either mildly or highly defended prey. Firstly, highly defended β should be rejected at lower energy reserves and toxin burdens than α (Figs 1B and 2B). Secondly, the prediction that predators should reject defended prey at lower energy reserves when detoxification is costly (κ = 1) compared to when it is not (κ = 0) (cf. Figs 2A, 2B, 1A and 1B), is most striking for the highly defended β (cf. Figs 2B and 1B). This is because the energetic benefit of eating toxic prey is reduced when detoxification is costly, and this reduction is relatively larger for the highly defended β than for α.

### Predator state may lead to undefended prey being avoided

The optimal strategy for a predator changes dramatically when both α and β are available compared to when there is only one defended prey type. When the cost of detoxification is negligible (κ = 0) then at high energy reserves and low toxin burden all prey should be rejected, including the undefended γ (Fig 1C). This is because the risk of starvation is lower when more prey are present, and so a predator should avoid a build-up of high energy reserves that would make it more vulnerable to predation. However, predators should eat the undefended prey (γ) when they have a high toxin burden (Fig 1C) and in cases where detoxification is costly (κ = 1, Fig 2C). This will ensure that the predator has sufficient energy reserves and avoids being close to starvation when their toxin burden prevents them from consuming defended prey.

### When toxic prey types are visually discriminable, the cost of detoxification has a marked effect on predators’ foraging strategies

Two clear effects of costly detoxification (cf. Figs 1C and 2C) are that (i) highly defended prey should only be eaten when energy reserves are very low, and (ii) mildly defended prey should only be rejected when predators are close to their maximum toxin burden or when they have very high energy reserves (Fig 2C). Essentially, when detoxification is costly, the optimal strategy for a predator is similar to when only α is available, because β is so costly it is almost always rejected (Fig 2C). In contrast, when detoxification is not costly, the highly defended β should be eaten in a larger area of the state-space, when energy reserves and the toxin burden are low (Fig 1C). This means that a predator is less likely to find itself having dangerously low energy reserves, and can therefore also afford to reject α at lower energy reserves and reduce its own predation risk by avoiding having high energy reserves (cf. Fig 1A and 1C).

### When defended prey types are visual mimics, detoxification cost has no effect on predators’ foraging strategies

When α and β are perfect mimics (Figs 1D and 2D), predators cannot preferentially choose α over β. As a consequence they are more likely to be in danger of starving and so they should attempt to store more energy by always eating the undefended γ. This is the case whether or not there is a detoxification cost (Figs 1D and 2D). Notably, the state-space at a high toxin burden, where all defended prey should be avoided, is wider when α and β are visual mimics compared to when they are distinguishable (cf. Figs 1D and 2D with Figs 1C and 2C). This is because a predator does not know the toxin content of the defended prey prior to ingestion and therefore should avoid all defended prey in order to not risk potentially ingesting a highly defended prey so close to its maximum tolerated toxin burden (Figs 1D and 2D). In this condition, detoxification cost has no effect on the strategy because the predator cannot preferentially eat only mildly defended prey, and so instead partly compensates for the extra cost by eating more γ.

### Impact on prey survival

**Fig 3:**
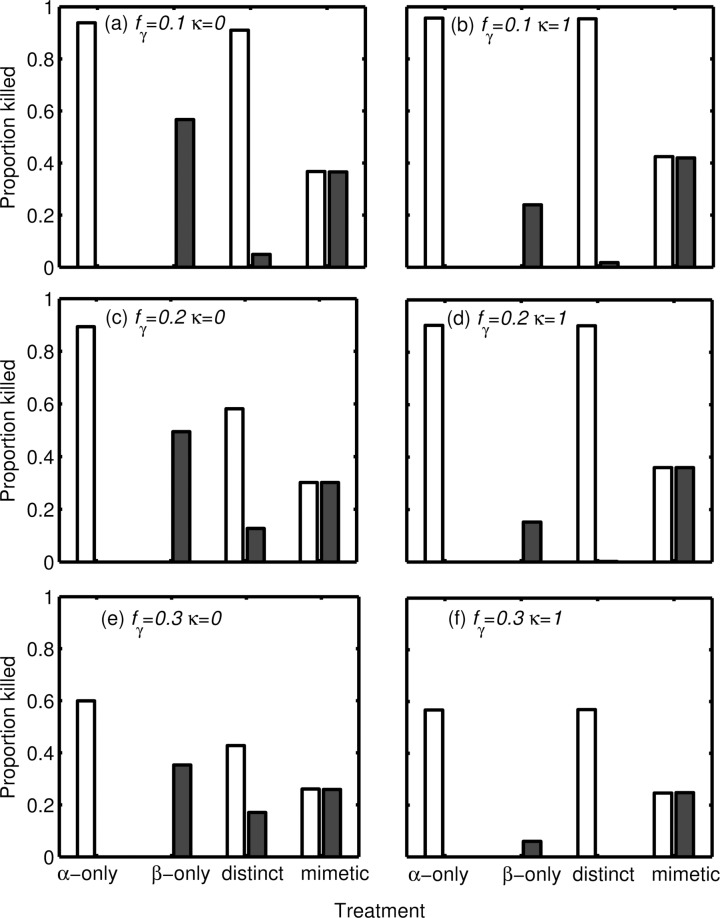
Mortality of mildly and highly defended prey types. Proportion of mildly defended α and highly defended β prey consumed by predators in the four experimental treatments (Mildly defended prey α alone; Highly defended prey β alone; α and β both present but distinguishably coloured; α and β both present and perfect mimics) for three values of the availability of alternative prey *f*γ (a, b) *f*γ = 0.1, (c, d) *f*γ = 0.2, (e, f) *f*γ = 0.3, and for whether detoxification is (a, c, e) cost-free (κ = 0) or (b, d, f) costly (κ = 1).

In Fig 3 we show how the cost of detoxification (κ), the abundance of alternative prey (γ), and mimicry affects selection on defended prey (α and β), through their impacts on predator decision-making.

### Increasing the abundance of alternative prey may decrease or increase the mortality of defended prey

For the visually mimetic α and β, the impact of increasing the abundance of alternative prey (γ) is similar whether or not detoxification is costly: the mortality of both α and β decreases with increasing abundance of γ (Fig 3), and the mortality of each prey type is lower than when presented alone. Both of these effects can be explained by the fact that there are now more nutrients available in the environment, and so the need to eat toxic prey decreases.

When α and β are visually distinguishable, however, the pattern is more complex. When detoxification is not costly (κ = 0), the mortality of each toxic prey type is lower than when it is present alone (Fig 3A, 3C and 3E). As expected, the mortality of α decreases with increasing γ, similarly to when it is the only toxic prey, however, the mortality of β increases with increasing γ, in contrast to when it is alone (Fig 3A, 3C and 3E). When there is no added cost associated with detoxification and the availability of γ increases, it becomes less important for predators to discriminate between α and β since energy reserves will be high. Therefore, the numbers of both prey types eaten will start to become more similar. Conversely, when γ decreases, the predator should discriminate between the unequally toxic α and β, when possible, and minimize its intake of β (Fig 3A).

**Fig 4:**
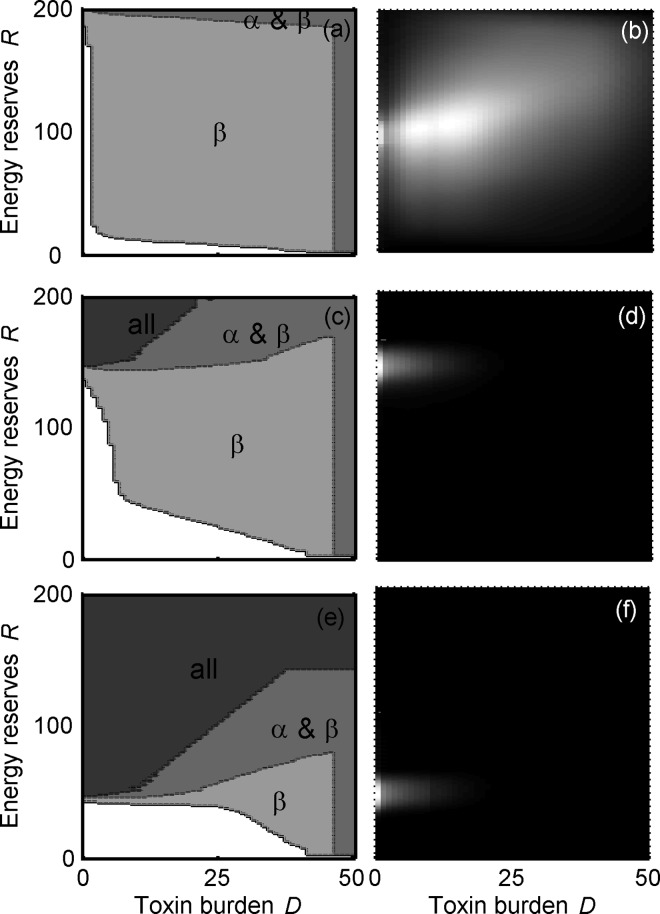
Impact of alternative prey on predator strategy and -state when defended prey are non-mimetic. Effect of availability of alternative prey (*f*γ) on predator strategy (a, c, e) and stationary distribution of predator state (b, d, f) in the non-mimetic treatment. Results are shown for three values of availability of alternative prey (a, b) γ = 0.1, (c, d) γ = 0.2, (e, f) γ = 0.3. The shaded areas show the states where: the predators reject all prey including alternative prey (black); reject only the mildly defended prey (pale grey); reject only the highly defended prey (intermediate grey) reject both defended prey (dark grey) (a, c, e). In the white areas, all prey are accepted. Distribution of predator states are shown from high (white) to zero (black) (b, d, f).

This effect can be seen in more detail from consideration of Fig 4, which shows not only the optimal strategy for the predator for three values of γ (left panels; Fig 4A, 4C and 4E), but also what area of the state-space predators tend to be found in, in terms of their energy reserves and toxin burden (right panels; Fig 4B, 4D and 4F). When the probability of encountering an undefended prey is low (γ = 0.1), there is a greater risk of starvation and so predators must accept α at most energy reserves and toxin burdens (Fig 4A). Therefore predators will often have an intermediate toxin burden and should avoid ingesting β in order to reduce their risk of reaching their maximum tolerated toxin burden (Fig 4B). When the availability of undefended prey is increased, however, the risk of starvation decreases and the optimal strategy for predators is now to maintain lower energy reserves, and reduce their own risk of predation. This means that predators are rarely in the state-space where eating a defended prey could kill them (Fig 4D and 4F), so it is less important for them to discriminate between α and β.

When detoxification is costly (κ = 1) the mortality of both α and β decreases with increasing γ (Fig 3B, 3D and 3F). This outcome for β, which is in contrast to when detoxification is not costly, can be explained by referring back to the equivalent state-space figure (Fig 2C) which shows that the optimal strategy for the predator in this condition is to only eat β when energy reserves are low, which may be the case when alternative prey are rare (γ = 0.1; Fig 3B). Notably, regardless of the frequency of alternative prey, the mortality of α is greater when detoxification is costly compared to when it is not (cf. Fig 3A, 3C and 3E and Fig 3B, 3D and 3F). This is because the predators need to increase their intake of the mildly defended prey in order to maintain energy reserves as they eat fewer β.

### The relative costs and benefits of mimicry to the model and mimic, respectively, depends on detoxification costs and alternative prey

The effects of mimicry on the mortality of α and β can be seen by comparing their mortalities when they are visually distinguishable compared to when they are mimetic (Fig 3). Regardless of the costs of detoxification and the frequency of alternative prey, α always benefits from mimicry whilst β suffers greater mortality when it has a visual, less toxic, mimic. This is because predators need to eat toxic prey in order to fulfil their energy and nutritional requirements but can no longer selectively ingest α.

However, the frequency of alternative prey and the costs of detoxification can affect the degree to which the mortalities of α and β differ between non-mimetic and mimetic conditions. When detoxification is not costly (κ = 0), increasing γ frequency reduces the benefits of mimicry to α along with the associated costs for β because predators become less discriminatory in the non-mimetic condition, and their behavior is more similar to when α and β mimic one another. It is not clear that this is the case when detoxification is costly (κ = 1). However, one intriguing finding is that the number of mimetic prey eaten (α plus β) is consistently higher when toxins are costly to detoxify than when they are not costly (Fig 3). This is because although the predators can afford to be at lower energy reserves due to the amount of available prey, when toxic prey are costly to metabolise the predators will require a greater food intake than when toxins are not costly for predators and thus they need to eat more toxic prey.

## Discussion

It is well established that the predation risk faced by predators themselves can have a significant effect on their foraging strategies (e.g. [53,54,55]). Our model predicts that balancing the risk of predation with the need to acquire nutrients (for maintanence, growth and reproduction) results in different physiological optima for the predator, depending on the availability of nutrients in the environment. This in turn affects predation on toxic prey, but we show that the precise nature of these effects depends on the physiological cost to predators associated with ingesting toxic prey. Below, we consider how the changing foraging strategies could impact on the evolutionary dynamics of different prey defences, as well as how strategies might be affected by increased nutritional needs.

### Highly toxic prey benefit from increased detoxification costs whilst mildly toxic prey do not

When there is only one toxic prey type in the environment, having a toxin that is metabolically costly for predators to detoxify is predicted to benefit highly toxic prey, which will suffer lower mortality. This is intuitive, but in contrast, mildly toxic prey suffer slightly greater mortality when their toxin is metabolically costly to predators. At first this appears counterintuitive, since we would expect that prey carrying a costly toxin would consistently be more highly defended than prey carrying a non-costly toxin. However, the underlying reason for this finding is the changing physiological optima of the predator. When mildly toxic prey are costly, predators should only reject them when they have high energy reserves or are near their maximum toxin burden. This is true when the toxin is not costly too, but in this case the predators are more likely to have high energy reserves than when the toxin is costly to metabolise. This in turn means that the mildly toxic prey will be eaten more often when the toxin is costly. Notably, in nature, there will be toxins that are costly to some predators but less so, or not at all, to others (e.g. [37,56,57]) and our model highlights the importance of considering not only the risk of injury or death that prey defences pose predators but also the costs imposed on predators through their need to mitigate these risks. This is analogous to the realisation that predators have strong non-lethal effects on their prey, such that the major ecological impact of predators on their prey may not be seen through direct mortality but through selection pressure for behavioural and/or physiological adaptations adopted by the prey to manage their risk of predation. This might include changes in the time of day at which prey are active, or changes in their diet [58].

### Unequally toxic prey mutually benefit from coexisting when their toxins are not costly to detoxify, but only highly toxic prey benefit when toxins are costly

When there are two unequally-toxic, non-mimetic prey types in the environment, we again find that the mortality of each of these will differ depending on whether detoxification is costly or not. When there are no costs associated with detoxification, both prey types are predicted to benefit from co-existing, compared to being the only defended prey type present. This is consistent with recent empirical findings from birds foraging on insect prey that support the theory of ‘toxin mutualism’ [15]. Toxin mutualism is where sympatric prey species benefit from using the same toxin even if they are visually distinguishable and unequally toxic, and it has been demonstrated in starlings (*Sturnus vulgaris*), with a recent experiment showing that they reduce their ingestion of two unequally toxic live prey types when they co-occur compared to when they don’t [15]. This is because predators can only ingest a certain amount of a toxin in any given time period (toxin saturation theory'; [59]), and it could be due to either physiological constraints or the excessive costs of processing toxins; for example, red knots (*Calidris canutus canutus*) appear to be toxin-limited when feeding on the toxic bivalve *Loripes lucinalis* [60]. However, the reduced mortality of both toxic prey types may also be explained by the increased amount of energy available to the predators when there are three prey types compared to only two.

In contrast, when detoxification is costly to predators, we find that sharing the same toxin is no longer mutually beneficial to the unequally toxic prey types. Instead, only the highly toxic prey type benefits from reduced mortality when existing alongside another, less toxic, prey type. The less toxic prey suffer the same level of mortality as when they are the only toxic prey type available. Overall, this suggests that in nature, selection pressures put on visually distinguishable defended prey will be very different depending on how costly the toxin is to a predator. This may be directly correlated with the concentration of the toxin and the energy required to detoxify it, or it could be relative to an individual predator’s ability to digest the toxin, which can be influenced by other components of a predators’ diet, or the state of the predator (e.g. [23,61]). Indeed, recent empirical findings suggest that predators in a poorer physiological state are less likely to discriminate between unequally defended non-mimics than those in a better state [15]. If a toxin carries no detoxification cost, and there is an abundance of prey in the environment, predators may also discriminate less between unequally toxic prey, and given the fact that chemical defences are often costly to sequester or synthesise (e.g. [62]) there may not be a strong selection for highly toxic prey under those circumstances. Notably however, when the abundance of alternative prey is low we find that predators will still discriminate between unequally toxic prey types.

### The cost of mimicry to the model is likely to vary as a result of seasonal and temporal changes to prey abundance and the risk of predation

Mildly toxic prey are predicted to enjoy their lowest mortality when they are visually mimetic to a more toxic model. This is because predators will reject the mildly toxic prey at lower toxin burdens when they cannot distinguish it from the highly toxic prey, in order to minimise the risk of ingesting too much toxin. In turn, mimicry is costly to the model, which suffers greater mortality compared to when it is visually distinguishable from the mildly toxic prey. This suggests that there may be selection for divergent evolution under these circumstances, as the model would benefit from evolving traits that made it distinguishable from the mimic [63–65]. However, the strength of these effects will also depend on the abundance of alternative prey and the predation risk facing the predator itself. This predation risk imposes a limit on the amount of energy the predator can keep in reserve, as seen in wild birds, where energy reserves negatively correlate with greater predation risk [66]. However, this limit can be increased when the risk of starvation is high, for example, birds increase their energy reserves in the winter when food is more scarce [66]. This means that highly-defended models, and less-defended mimics, will be consumed more frequently when alternative prey are rare,. Further, since the relative cost of mimicry to the model increases as the abundance of alternative prey decreases, there may also be a stronger selection for divergent evolution. This marked impact of the availability of alternative prey, and how this affects predator state, highlights that the selection pressures facing toxic prey, and in turn mimicry dynamics, is likely to vary as a result of seasonal and temporal changes in relative species abundances. At times when prey are scarce, or difficult to find, toxic prey are likely to be predated upon more frequently as they provide much-needed nutrients [20,21,67]. It has for instance been reported that starlings will increase their attacks on prey that they know to be toxic when undefended prey are better concealed [67]. However, it is important to consider that at such times when prey are harder to find the predation risk for the predator itself may also be increased, which can impact on how much energy, in the form of body mass, a predator is willing or able to store [45,47]. Further investigations into the impact of variable predation rates on predators could help us better understand how selection on prey defences might vary not only as a result of variability in the abundance of alternative prey in the environment (see also [21]), but also the stresses faced by the predator itself.

### The mortality of both mimics and models is greater when the toxin is costly for a predator to metabolise

As when the two toxic prey types are visually distinguishable (see above), the cost of the toxin once again has a significant effect on the survival of the prey, but (perhaps surprisingly) our model predicts that the mortality of both mimics and models is greater when the toxin is costly to metabolise, compared to when it isn’t. This can, however, be explained by the predators needing to eat more of all prey types in order to have sufficient energy reserves to digest the more costly toxin. A similar effect is seen in nectivorous birds, which consume more toxins in nectar when the nectar is dilute compared to when it is concentrated in order to maintain their required energy intake [39]. This again suggests that the efficacy of a toxin, in terms of increasing prey survival, will not necessarily correlate with how costly the toxin is to metabolise by predators. Instead it will also likely be influenced by the state of the predator, something which was also highlighted in a recent model investigating how prey nutrition and toxin dose impact on predator foraging strategies ([38], see also [68]). It seems that there is likely to be a complex interrelationship between predator state and how costly or beneficial a toxic prey item is perceived to be, which in turn may determine the dynamics of mimicry systems in the wild. Whilst the presence of less toxic mimics in the environment is generally considered costly to their more highly toxic co-mimic (compared to when they are the only toxic prey around), by increasing the number of attacks by predators and decreasing their survival, we find that this is only the case when the toxin is costly to metabolise. When the toxin is not costly, both prey species benefit from increased survival when they are visual mimics, compared to when they are the only toxic prey type in the environment. These alternative outcomes come about as a result of the state-space in which the predator most often finds itself in the two different scenarios: when toxins are costly predators will be using up more energy to detoxify and so they will spend more time closer to starvation and in turn need to increase their prey intake; but when toxins are not costly, predators are rarely close to starvation and so can decrease their intake of toxic prey. This effect of predator state and detoxification costs, combined with the use of different methodologies and predator species, could potentially explain some of the variability found amongst empirical findings into the dynamics between unequally toxic co-mimics, where in some cases they benefit mutually from reduced predation but in others the relationship is a parasitic one [15–18].

### Non-toxic alternative prey could benefit from reduced mortality, when in the presence of toxic prey types, as a result of predation risks facing the predator itself

Whilst it is often presumed that non-toxic prey in the same environment as toxic prey should always be eaten when found (e.g. [14,51,69]), our model predicts that this is not necessarily the case when the predator’s own predation risk is taken into account. In fact, if there is no significant cost to predators of detoxification then we find that the presence of two visually distinguishable toxic prey species may be beneficial to non-toxic prey. This results from there being an abundance of distinguishable prey in the environment, which means that there is no risk of starvation and no risk of predators eating highly toxic prey in error. Consequently the predator should adopt a ‘mass regulation strategy’, whereby it maintains a lower body mass in order to increase its chances of escaping predatory attacks (e.g. [44,47]). However, this potential benefit to non-toxic prey only occurs when the predator has a low toxin burden and high energy reserves, and it disappears when the digestion of toxic prey carries a metabolic cost. This is because predators now should adopt a strategy of avoiding toxic prey and only eating them at low energy reserves: predators need to eat all non-toxic prey that they encounter to decrease their risk of starvation. Overall, these predictions suggest that the metabolic cost associated with digesting toxic prey will affect selection on alternative non-toxic prey in the environment, as predators faced with more costly toxic prey shift their physiological optimum. Relatively few predators are free from interspecific-predation sothis behaviour is likely to be ubiquitous.

## Conclusions

Predator decision-making has a significant impact on the survival of both toxic and non-toxic prey in the environment and so on the dynamics of mimetic relationships between unequally toxic prey. In nature, aposematic prey will vary in their toxin content both within and between species (e.g. [6,70,71]), and the risk of a predator being predated will also varies [45] so the dynamics of mimetic relationships will be inconsistent across time and space In addition, whilst we have investigated the effects of predation risk increasing with energetic reserves, since this is both intuitive and has empirical support (e.g. [48,49,50]), any factors that limit energy storage would have the same effect, such as the metabolic costs or carrying fat and/or selective pressures associated with activities other than foraging [72]. Notably, in order for such variables to be incorporated into our model and give realistic predictions we first need to learn more about the interrelationship between these variables and energetic reserves in natural predator-prey systems. It remains clear, however, that our results potentially have broader implications for any tri-trophic foraging system where predators impact on foragers’ decisions to include or reject toxic foods.

## Supporting Information

S1 Appendix Complete description of the model implementation. (DOCX)
