# Supplementary material for: The Impact of Detoxification Costs and Predation Risk on Foraging: Implications for Mimicry Dynamics
Source: PLoS One. 2017 Jan 3;12(1):e0169043. doi: 10.1371/journal.pone.0169043 (PMC5207405; doi:10.1371/journal.pone.0169043)
Supplement: S1 Appendix — (DOCX) [file pone.0169043.s001.docx]

**S1 Appendix**

Here, we provide a complete description of the model implementation that follows standard dynamic programming methods [[1](#_ENREF_1)]. We characterize the current physiological state of an individual predator as combinations of integer values of energy reserves and toxin burden (*R, D*). The optimal strategy is the set of decisions δ(*R, D*) [see equation (2) in the main text] that minimize the long-term rate of mortality. Mortality can occur from three events: (i) energy reserves reach zero (*R=*0): the animal has starved to death; (ii) toxin burden reaches the maximum (*D=Dmax*): the animal has been poisoned; (iii) a successful attack by a predator of the focal predator.

At each time-step the predator stochastically encounters nothing (denoted ω) or a prey type *p*, so$p\in\{\alpha,\beta,\gamma,\omega\}$. The probabilities of each of these four events (encountering either an α, β or γ individual or nothing ω) are determined by the population densities of the three prey types *f*_α_, *f*_β_, *f*_γ_. Each prey type also has a conspicuousness *c_i_* controlling the probability that a prey item of that type is detected if it is encountered (i.e. in the vicinity of) by the predator. Thus, the detection rate of each prey is *f_p_c_p_* where *p* is α, β or γ. The probability of not detecting any prey is given by:

$\omega=\sum_{p\in\{\alpha,\beta,\gamma\}} 1-f_{p}c_{p}$ (A1)

All population densities are scaled to ensure that all these probabilities sum to unity.

If the predator has any toxins, it removes them at a rate of one unit per time step at an energetic cost κ. For computational reasons, we assume that the basal energetic cost of metabolism may vary, being ρ*_i_* on each time step, where *i*=1 or 2 and ρ_1_=ρ-0.5 or ρ_2_=ρ+0.5

If the predator eats a prey item of type *p*, its states at the next time step are given by:

$\begin{matrix} R'=R+r_{p}-\rho_{i}-\kappa\text{ if }D>0 \\ R'=R+r_{p}-\rho_{i}\text{ if }D=0 \end{matrix}$ (A2a)

and

$\begin{matrix} D'=D+d_{p}-1\text{ if }D>0 \\ D'=D+d_{p}\text{ if }D=0 \end{matrix}$ (A2b)

Where the metabolic cost of being alive is unity. If the predator does not eat a prey item, *r_p_* and *d_p_* take the value of zero in equation A2.

Given these ingredients, the dynamic programming operator *T** can be expressed as follows. Let *V* be a function, $V\left(R,D\right)$, of energy reserves *R* and toxin burden *D*  satisfying $V\left(0,D\right)=0$and $V\left(R,D=D_{\max}\right)=0$. Then $T *V$is a new function of reserves and environmental conditions that satisfies $(T*V)\left(R,D\right)=\max_{\delta} H(R,D;\delta,V)$ for *R* > 0 and *D*<*D_max_*, where

$H(R,D;\delta,V)=(1-M)\sum_{j=1}^{4} p_{j}\sum_{i=1}^{2} \frac{1}{2}V(R',D')$ (A3)

in which *R’* and *D’* are given by equation (A2) and whether the predator accepts the prey type *p* (equation 2) and *M* is given by equation (1) in the main text.

To find the optimal strategy, we define a sequence of functions $V_{0}, V_{1}, V_{2},\cdots$ iteratively as follows. Initially set $V_{0}\left(0,D\right)=0$ for all *D*; $V_{0}\left(R,D_{\max}\right)=0$ for all *R* and $V_{0}\left(R,D\right)=1$ otherwise. Given $V_{k}$, set $V_{k+1}=T *V_{k}$. Then the sequence of functions converges pointwise to a limit $V *=\lim_{k\to\infty} V_{k}$ [[2](#_ENREF_2)]. Convergence was judged to have occurred when $\left|V_{k+1}-V_{k}\right|<10^{-6}$, which typically happened within 200 iterations. Any strategy *f** satisfying .

When an *α* or *β* individual is encountered a misidentification occurs with probability ε. When α and β are distinguishable there is no error in the predator’s recognition (ε=0). For perfect mimicry (ε=1) selectivity between α and β are simply not available to the predator (δ ≠ 1 or 2). Note that we do not allow for any error in attacking prey γ: they are assumed to be conspicuous and distinguishable from both α and β.

We run stochastic simulations with *N* predators following the optimal strategy δ*(*R,D*) and assuming that the prey population is infinite such that prey proportions are constant even with depletion by the predators. We ignore the decisions made in the first 500 time steps in order to not consider decisions before the predator state distribution has converged. The simulation provides the number of times that each prey type survives (*S_p_*) or does not survive (*K_p_*) an encounter with a predator.

**Appendix References**

1. Houston AI, McNamara JM. Models of adaptive behaviour: an approach based on state. Cambridge, UK: Cambridge University Press. 1999.

2. Puterman ML. Markov decision processes: Discrete stochastic dynamic programming. New Jersey: Wiley. 2005.
